# Modified median quartile double ranked set sampling for estimation of population mean

**DOI:** 10.1016/j.heliyon.2024.e34627

**Published:** 2024-07-14

**Authors:** Muhammad Ahmed Shehzad, Anam Nisar, Aamna Khan, Walid Emam, Yusra Tashkandy, Haris Khurram, Isra Al-Shbeil

**Affiliations:** a Department of Statistics, Bahauddin Zakariya University, Multan, Pakistan; b Department of Statistics and Operations Research, Faculty of Science, King Saud University, P.O. Box 2455, Riyadh, 11451, Saudi Arabia; c Department of Sciences & Humanities, National University of Computer and Emerging Sciences, Chiniot-Faisalabad Campus, Pakistan; d Department of Mathematics, Faculty of Science, The University of Jordan, Amman, 11942, Jordan; e Department of Mathematics and Statistics, University of Ottawa, Ottawa, ON, K1N 6N5, Canada; f Department of Mathematics and Computer Science, Faculty of Science and Technology, Prince of Songkla University, Pattani Campus, Pattani, Thailand

**Keywords:** Ranked set sampling (RSS), Median double ranked set sampling (MDRSS), Quartile double ranked set sampling (QDRSS), Extreme double ranked set sampling (EDRSS), Population mean

## Abstract

Environmental monitoring and assessment aim to gather data economically, without bias, using efficient and cost-effective sampling methods. One such traditional method is Ranked Set Sampling (RSS), often employed to achieve observational economy. This article introduces an innovative two-stage sampling approach for ranked set sampling (RSS) to get a more precise estimate of the population mean. Modified Median Quartile Double Ranked Set Sampling (MMQDRSS) highlights the ranked base technique's potential as a cost-effective sampling method. To evaluate the performance of the proposed estimator by using real-life data and conducting a simulation study to compare the relative efficiency of the proposed estimator with some existing methods.

## 1 Introduction

Ranked set sampling (RSS) serves as an economical and effective substitute for simple random sampling (SRS) in particular scenarios. When measuring certain things is difficult or expensive, but arranging them based on the variable of interest is simple and inexpensive, RSS is a good choice to measure the actual desired results. It has been shown that it and its forms are better at predicting many population factors than SRS. RSS is a way to pick samples that makes statistical predictions more accurate by looking at the order or ranking of events in a sample instead of just their values. In some situations, RSS can be used instead of SRS because it is cheaper and works just as well. When it's hard or expensive to measure certain things but simple and cheap to arrange them based on the variable of interest, RSS is a good choice. It has been shown that it and its forms are better at predicting many population factors than SRS. RSS is a way to pick samples that makes statistical predictions more accurate by looking at the order or ranking of events in a sample instead of just their values [1].Random selection is done in a different way with RSS. Each observation is viewed as separate and equally important in standard random sampling. However, RSS takes advantage of the natural order or ranking of the data. This makes RSS a more valuable method for producing more accurate estimates than SRS. Modified form of Extreme RSS (ERSS) [2,3] introduced the Ranked Set Sampling Scheme, which is a novel sampling technique. He observed that the simple random sampling (SRS) technique yielded more accurate estimates. To illustrate this procedure [4], provided the required computational results for RSS. They found that the RSS method gives a more accurate estimate of the population mean with less variation, similar to how the SRS method gives an estimate of the sample mean's variance. Proposed [5] occasional ranking errors may occur. They illustrated how inaccuracies in ranking lead to efficiency losses. The Proposed concomitant variable as a tool to aid in the ranking process and generate ranked set data [6]. In order to draw conclusions regarding the variance and correlation coefficient of the population, she has also examined the ranked set sample method. Instead of using subjective opinion to rank the elements, we used the auxiliary variable in this case. The objectivity of this modification as a population mean estimator. Given that the parent distribution is symmetric, they produced more accurate results than the RSS estimator. The ratio within RSS was analyzed, and it was corroborated that ranking based on the independent variable X is more effective than ranking based on the variable under study Y. This led to the proposal of an innovative sampling method called median ranked set sampling (MRSS) [7]. They have demonstrated the objectivity of this modification as a population mean estimator. The quartile-ranked set sample (QRSS), a newly modified variation of the RSS [2]. In the research, took into account a few distributions and discovered that for mean estimation, QRSS estimates are more accurate than SRS. Modified forms, such as MRSS and QRSS, are used for the estimation based on parameters [2,8], an extended RSS into the DRSS and QDRSS systems, and so on, to achieve more effective population mean estimation than the standard RSS method [9]. By altering the RSS [10,11], proposed Extreme DRSS and Median DRSS to boost the effectiveness of the population mean estimator. PPS-based double sampling approaches better estimate parameters with extreme values when data is scarce or nonexistent, distributing the value across multiple ranges of unit sizes. This is supported by outlier observations in the population [12]. Estimate the central tendency using two-phase and simple random sampling with auxiliary variables. Compare the mathematical expressions of the proposed estimators for the mean squared errors with Naik and Gupta's mean estimator and find that the proposed estimator performs better on a large number of real-life datasets [13]. New exponential-type estimators based upon two auxiliary variables for population mean estimation and elaborating their efficiency for simple random as well as stratified random sampling [14]. Modified median ranked set sampling (MMRSS) [15] and median quartile double ranked set sampling (MQDRSS) [16] methods introduced. In fields such as environmental, ecological, and agricultural studies, a well-designed and efficient sampling scheme is of paramount importance. Thus, this article introduces a novel and more efficient scheme termed Modified Median Quartile DRSS (MMQDRSS) for population mean estimation. MMQDRSS offers an unbiased population mean estimator under symmetrical distributions, consistently outperforming SRS in terms of mean and variance estimators. Through comprehensive ranking-based simulations across symmetrical and non-symmetrical distributions, the MMQDRSS is evaluated alongside existing DRSS schemes and the SRS scheme.

### 1.1 Ranked set sampling

RSS is considered a cost-effective and efficient alternative to employing simple SRS. The concept of RSS to estimate pasture production averages [8]. Apart from the conventional SRS method, RSS is recognized as a valuable sampling approach for achieving precise population mean estimates. The process begins with a random selection of $m^{2}$ units from the target population. Each set is then allocated m units from this selection. These units are ranked either in ascending or descending order using visual or auxiliary variable methods. Next, from these ranked sets, one unit from each set is chosen in a systematic manner, starting with the highest-ranked unit in the first set and continuing until the $m^{th}$ highest-ranked unit in the $m^{th}$ set is selected. This process is repeated r times to obtain a sample size of n=mr.

The population's RSS mean estimator is,

$${\bar{W}}_{\left(RSS\right)}=\frac{1}{mr}\sum_{i=1}^{mr}{W\;}_{i\left(i\right)}$$

With variance,

$$Var\left(Z_{\left(RSS\right)}\right)=\frac{\sigma^{2}}{mr}-\frac{1}{rm^{2}}\sum_{i=1}^{m}{\left(\mu_{\left(i\right)}-\mu \right)}^{2}$$

### 1.2 Extreme double ranked set sampling

An amendment to DRSS is proposed to obtain an efficient sampling scheme [10,11] to estimate the population mean known as Extreme RSS (EDRSS). In this method, similar to the DRSS. In the first step, $m^{3}$ units are randomly chosen from the underlying population. In the second step, distribute these $m^{3}$ sampling units divided into m sets with same set size $m^{2}$ at random. For each set, use RSS. of $m^{2}$ units and obtain m ranked-set samples of m size each. In the third and final step to get the EDRSS, utilize ERSS on m using ranked-set samples to choose a sample of the desired size m. The whole methodology can be reproduced in r. The number of cycles required to determine the complete sample size n=mr.

Population mean estimator along with variance based on EDRSS for a single cycle is presented as (for even):

$${\bar{W}}_{\left(EDRSS\right)e}=\frac{1}{mr}\left[\sum_{i=1}^{q_{1}}W_{1\left(1\right)}^{\left(i\right)\left(1\right)}+\sum_{i=\left(m/2\right)+1}^{q}W_{q\left(q\right)}^{\left(i\right)\left(q\right)}\right]\text{,}$$

And variance,

$$Var\left({\bar{W}}_{\left(EDRSS\right)e}\right)=\frac{1}{m^{2}}\left[\begin{array}{c} \sum_{i=1}^{q_{1}}Var\left(W_{1\left(1\right)}^{\left(i\right)\left(1\right)}\right)\\ +\sum_{i=\left(q_{1}\right)+1}^{m}Var\left(W_{q\left(q\right)}^{\left(i\right)\left(q\right)}\right) \end{array}\right]\text{,}$$

For odd,

$${\bar{W}}_{\left(EDRSS\right)o}=\frac{1}{mr}\left[\sum_{i=1}^{\left(m-1\right)/2}W_{1\left(1\right)}^{\left(i\right)\left(1\right)}+W_{q_{2}\left(q_{2}\right)}^{\left(q_{2}\right)\left(q_{2}\right)}+\sum_{i=q_{2}+1}^{q}W_{q\left(q\right)}^{\left(i\right)\left(q\right)}\right]\text{,}$$

And the respective variance is,

$$Var\left({\bar{W}}_{\left(EDRSS\right)o}\right)=\frac{1}{m^{2}}\left[\begin{array}{c} \sum_{i=1}^{\left(m-1\right)/2}Var\left(W_{1\left(1\right)}^{\left(i\right)\left(1\right)}\right)+Var\left(W_{q_{2}\left(q_{2}\right)}^{\left(q_{2}\right)\left(q_{2}\right)}\right)\\ +\sum_{i=q_{2}+1}^{m}Var\left(W_{l\left(l\right)}^{\left(i\right)\left(l\right)}\right) \end{array}\right]\text{.}$$

where, q=m, $q_{1}=m/2$ and $q_{2}=\left(m+1\right)/2$.

### 1.3 Median double ranked set sampling

To further improve the efficiency of the DRSS sampling scheme for estimating the population mean, a new modification called Median DRSS is proposed (MDRSS) [10,11]. In this modification which based on DRSS and EDRSS, $m^{3}$ units are randomly drawn from the population. Then, distribute these $m^{3}$ units at random into m sets with the same set of size $m^{2}$. Apply RSS on each set of $m^{2}$ units and obtain m ranked-set samples of m size each. The final MDRSS estimate is obtained by using MRSS to select a sample of size m from the ranked-set samples. This whole process can be reprocessed in the form of r cycles for selecting complete sample size n=mr. A method for estimating the population mean and its variance based on EDRSS for one cycle is follows as:

For even,

$${\bar{W}}_{\left(MDRSS\right)e}=\frac{1}{mr}\left[\sum_{i=1}^{q1}W_{q1\left(q1\right)}^{\left(i\right)\left(q1\right)}+\sum_{i=\left(q1\right)+1}^{m}W_{q2\left(q2\right)}^{\left(i\right)\left(q2\right)}\right]\text{,}$$

Variance is,

$$Var\left({\bar{W}}_{\left(MDRSS\right)e}\right)=\frac{1}{m^{2}}\left[\begin{array}{c} \sum_{i=1}^{q1}Var\left(W_{q1\left(q1\right)}^{\left(i\right)\left(q1\right)}\right)\\ +\sum_{i=\left(m/2\right)+1}^{m}Var\left(W_{q2\left(q2\right)}^{\left(i\right)\left(q2\right)}\right) \end{array}\right]\text{,}$$

For odd,

$${\bar{W}}_{\left(MDRSS\right)o}=\frac{1}{mr}\left[\sum_{i=1}^{m}W_{q_{2}\left(q_{2}\right)}^{\left(i\right)\left(q_{2}\right)}\right]\text{,}$$

And variance,

$$Var\left({\bar{W}}_{\left(MDRSS\right)o}\right)=\frac{1}{m^{2}}\left[\sum_{i=1}^{m}Var\left(W_{q_{2}\left(q_{2}\right)}^{\left(i\right)\left(q_{2}\right)}\right)\right]\text{,}$$

where, $q_{1}=m/2$ and $q_{2}=\left(m+1\right)/2\text{.}$.

### 1.4 Quartile double ranked set sampling

Quartile DRSS (QDRSS) is a proposed modification to the DRSS sampling scheme that aims to improve efficiency in estimating the population mean [5]. In this modification, based on the basic DRSS, EDRSS, and MDRSS, units were chosen $m^{3}$ randomly from the underlying population. Then, disperse these $m^{3}$ units into m sets at random with same set size $m^{2}$. Utilize RSS on each group of $m^{2}$ units and obtain m ranked-set samples with an m size. In the final stage, to get the QDRSS, use QRSS on m ranked-set samples to select a size m. This whole procedure can be utilized in the form of m cycles for selecting a complete sample size n=mr. The following is a QDRSS-based a population mean and variance estimator for one cycle:

For even,

$$W_{\left(QDRSS\right)e}=\frac{1}{mr}\left[\sum_{i=1}^{m/2}W_{q_{1}\left(q_{1}\right)}^{\left(i\right)\left(q_{1}\right)}+\sum_{i=\left(m/2\right)+1}^{m}W_{q_{3}\left(q_{3}\right)}^{\left(i\right)\left(q_{3}\right)}\right]\text{,}$$

And variance,

$$Var\left({\bar{W}}_{\left(QDRSS\right)e}\right)=\frac{1}{m^{2}}\left[\begin{array}{c} \sum_{i=1}^{m/2}Var\left(W_{q_{1}\left(q_{1}\right)}^{\left(i\right)\left(q_{1}\right)}\right)\\ +\sum_{i=\left(m/2\right)+1}^{m}Var\left(W_{q_{3}\left(q_{3}\right)}^{\left(i\right)\left(q_{3}\right)}\right) \end{array}\right]\text{,}$$

For odd,

$${\bar{W}}_{\left(QDRSS\right)o}=\frac{1}{mr}\left[\sum_{i=1}^{\left(m-1\right)/2}W_{q_{1}\left(q_{1}\right)}^{\left(i\right)\left(q_{1}\right)}+Y_{q_{2}\left(q_{2}\right)}^{\left(q_{2}\right)\left(q_{2}\right)}+\sum_{i=q_{2}+1}^{m}W_{q_{3}\left(q_{3}\right)}^{\left(i\right)\left(q_{3}\right)}\right]\text{,}$$

And variance,

$$Var\left({\bar{W}}_{\left(QDRSS\right)o}\right)=\frac{1}{m^{2}}\left[\begin{array}{c} \sum_{i=1}^{\left(m-1\right)/2}Var\left(W_{q_{1}\left(q_{1}\right)}^{\left(i\right)\left(q_{1}\right)}\right)+Var\left(W_{q_{2}\left(q_{2}\right)}^{\left(q_{2}\right)\left(q_{2}\right)}\right)\\ +\sum_{i=q_{2}+1}^{m}Var\left(W_{q_{3}\left(q_{3}\right)}^{\left(i\right)\left(q_{3}\right)}\right) \end{array}\right]\text{.}$$

where, $q_{1}=\left(m+1\right)/4$, $q_{2}=\left(m+1\right)/2$ and $q_{3}=\left(3\left(m+1\right)/4\right)$.

### 1.5 Proposed modified median quartile double ranked set sampling (MMQDRSS)

The Modified Median Quartile DRSS (MMQDRSS) is a two-stage sampling scheme in which MRSS is used at the first stage while QRSS at the second stage to draw a more representative sample of m units. It is an efficient sampling strategy, and it would be much better if the ranking mechanism of the feature of interest occurred at no cost. The proposed ranked-based MMQDRSS technique is presented in the steps below:

Step 1 Draw m^3^ units at random derived from the target population and divide them into m sets of m units.
Step 2 Using visual examination or any other cost-effective method, rank the units within each set.
Step 3 Using the MRSS procedure, select c (c ≤ m) units from the c sets, where c denotes the sets in which the median-ranked unit will be identified.
Step 4 Using the standard ERSS procedure, select the remaining (m-c) units from the (m-c) sets.
Step 5 Rank each unit select MRSS and ERSS from Steps 3 and 4, and then use QRSS 1 s stage to select c (c ≤ m) units from the c sets and use ERSS procedure, select the remaining (m-c) units from the (m-c) sets to choose an improved DRSS (MMQDRSS) of size m for the actual measurement.
Step 6 Steps 1 through 5 should be repeated r times to get a sample of size m for the actual measurement.
Step 7 For c = 0, the proposed design is identical to ERSS, and for c = m, it is equivalent to MRSS and QRSS. As a result, the design that is suggested is a subset of the MRSS, QRSS, and ERSS designs.

### 1.6 Example of MMQDRSS

For *m* = 7, *c* = 3, and m-c = 4, the MMQDRSS can be selected as follows.

To select an MMQDRSS of size n = 7 for r = 1 (m = 7, c = 3), identify m = 343 (7 sets of 49 sampling units each). Consider, $Z_{i\left(j\right)k}$ become $j^{th}$ the lowest ranked unit from $i^{th}$ subsection of the set $k^{th}$, in which i,j,k=1,2,3,…,7. Order the units in each subset of the five sets based on the variable being studied.

(G) $$\begin{array}{c} \begin{array}{c} \left[\begin{array}{c} W_{11\left(1\right)}W_{12\left(1\right)}W_{13\left(1\right)3\;}W_{14\left(1\right)}W_{15\left(1\right)}W_{16\left(1\right)}W_{17\left(1\right)}\\ W_{21\left(1\right)}W_{22\left(1\right)}W_{23\left(1\right)}W_{24\left(1\right)}W_{25\left(1\right)}W_{26\left(1\right)}W_{27\left(1\right)}\\ W_{31\left(1\right)}{\;W}_{32\left(1\right)}W_{33\left(1\right)}{\;W}_{34\left(1\right)}W_{35\left(1\right)}W_{36\left(1\right)}W_{37\left(1\right)}\\ W_{41\left(1\right)}W_{42\left(1\right)}W_{43\left(1\right)}W_{44\left(1\right)}W_{45\left(1\right)\;}W_{46\left(1\right)}W_{47\left(1\right)}\\ W_{51\left(1\right)}W_{52\left(1\right)}W_{53\left(1\right)}{W\;}_{54\left(1\right)\;}W_{55\left(1\right)}W_{56\left(1\right)}W_{57\left(1\right)}\\ W_{61\left(1\right)}W_{62\left(1\right)}W_{63\left(1\right)}W_{64\left(1\right)\;}W_{65\left(1\right)}W_{66\left(1\right)}W_{67\left(1\right)}\\ W_{71\left(1\right)}W_{72\left(1\right)}W_{73\left(1\right)}W_{74\left(1\right)\;}{W\;}_{75\left(1\right)}W_{76\left(1\right)}W_{77\left(1\right)} \end{array}\right]\\ \left[\begin{array}{c} W_{11\left(2\right)}W_{12\left(2\right)}W_{13\left(2\right)}W_{14\left(2\right)}W_{15\left(2\right)}W_{16\left(2\right)}W_{17\left(2\right)}\\ W_{21\left(2\right)}W_{22\left(2\right)}W_{23\left(2\right)}W_{24\left(2\right)}W_{25\left(2\right)}W_{26\left(2\right)}W_{27\left(2\right)}\\ W_{31\left(2\right)}{\;W}_{32\left(2\right)}W_{33\left(2\right)}{\;W}_{34\left(2\right)}W_{35\left(2\right)}W_{36\left(2\right)}W_{37\left(2\right)}\\ {W_{41\left(2\right)}\;W}_{42\left(2\right)}W_{43\left(2\right)}W_{44\left(2\right)}W_{45\left(2\right)\;}W_{46\left(3\right)}W_{47\left(2\right)}\\ W_{51\left(2\right)}W_{52\left(2\right)}W_{53\left(2\right)}W_{54\left(2\right)\;}W_{55\left(2\right)}W_{56\left(2\right)}W_{57\left(2\right)}\\ W_{61\left(2\right)}W_{62\left(2\right)}W_{63\left(2\right)}W_{64\left(2\right)\;}W_{65\left(2\right)}W_{66\left(2\right)}W_{67\left(2\right)}\\ W_{71\left(2\right)}W_{72\left(2\right)}W_{73\left(2\right)}W_{74\left(2\right)}{W\;}_{75\left(2\right)}W_{76\left(2\right)}W_{77\left(2\right)} \end{array}\right]\\ \vdots \end{array}\\ \left[\begin{array}{c} W_{11\left(7\right)}W_{12\left(7\right)}W_{13\left(7\right)}W_{14\left(7\right)}W_{15\left(7\right)}W_{16\left(7\right)}W_{17\left(7\right)}\\ W_{21\left(7\right)}W_{22\left(7\right)}W_{23\left(7\right)}W_{24\left(7\right)}W_{25\left(7\right)}W_{26\left(7\right)}W_{27\left(7\right)}\\ W_{31\left(7\right)}{\;W}_{32\left(7\right)}W_{33\left(7\right)}{\;W}_{34\left(7\right)}W_{35\left(7\right)}W_{36\left(7\right)}W_{37\left(7\right)}\\ {W_{41\left(7\right)}\;W}_{42\left(7\right)}W_{43\left(7\right)}W_{44\left(7\right)}W_{45\left(7\right)}W_{46\left(7\right)}W_{47\left(7\right)}\\ W_{51\left(7\right)}W_{52\left(7\right)}W_{53\left(7\right)}W_{54\left(7\right)\;}W_{55\left(7\right)\;}W_{56\left(7\right)}W_{57\left(7\right)}\\ W_{61\left(7\right)}W_{62\left(7\right)}W_{63\left(7\right)}W_{64\left(7\right)}W_{65\left(7\right)}W_{66\left(7\right)}W_{67\left(7\right)}\\ W_{71\left(7\right)}W_{72\left(7\right)}W_{73\left(7\right)}W_{74\left(7\right)\;}{W\;}_{75\left(7\right)}W_{76\left(7\right)}W_{77\left(7\right)} \end{array}\right] \end{array}$$

Then, in each set, choose the center units in which blocks, and the units used for sampling in every set are displayed in rows from eq (G), as shown below:

$$\left[\begin{array}{c} W_{11\left(1\right)}W_{12\left(1\right)}W_{13\left(1\right)}W_{14\left(1\right)}W_{15\left(1\right)}W_{16\left(1\right)}W_{17\left(1\right)}\\ W_{21\left(2\right)}W_{22\left(2\right)}W_{23\left(2\right)}W_{24\left(2\right)}W_{25\left(2\right)}W_{26\left(2\right)}W_{27\left(2\right)}\\ W_{31\left(3\right)}W_{32\left(3\right)}W_{33\left(3\right)}W_{34\left(3\right)}W_{35\left(3\right)\;}W_{36\left(3\right)}W_{37\left(3\right)}\\ W_{41\left(4\right)}W_{42\left(4\right)}W_{43\left(4\right)}W_{44\left(4\right)}W_{45\left(4\right)}W_{46\left(4\right)}W_{47\left(4\right)}\\ W_{51\left(5\right)}W_{52\left(5\right)}W_{53\left(5\right)}W_{54\left(5\right)}W_{55\left(5\right)}W_{56\left(5\right)}W_{57\left(5\right)}\\ W_{61\left(6\right)}W_{62\left(6\right)}W_{63\left(6\right)}W_{64\left(6\right)}W_{65\left(6\right)}W_{66\left(6\right)}W_{67\left(6\right)}\\ W_{71\left(7\right)}W_{72\left(7\right)}W_{73\left(7\right)}W_{74\left(7\right)}W_{75\left(7\right)}W_{76\left(7\right)}W_{77\left(7\right)} \end{array}\right]$$

Without determining the real measurement of these sub-section units, sort the number of each subsection in the preceding set once more. Sub-sequent, select the ${\left(\frac{m+1}{4}\right)}^{th}$ a ranked unit (in boxes), $W_{i\left(1:3\right)}^{*}$ to the $i^{th}$ sub-section (i=1,2,3) and select extremes unit of rank (in boxes), i.e., $W_{i\left(4:7\right)}^{*}$ to $i^{th}$ sub-section (i=4,5,6,7) the actual estimation is listed below:

$$\left[\begin{array}{c} W_{1:1}^{*}W_{1:2}^{*}\;W_{1:3}^{*}W_{1:4}^{*}W_{1:5}^{*}W_{1:6}^{*}W_{1:7}^{*}\\ W_{2:1}^{*}W_{2:2}^{*}W_{2:3}^{*}W_{2:4}^{*}{W_{2:5}^{*}W_{2:5}^{*}W}_{2:7}^{*}\\ W_{3:1}^{*}W_{3:2}^{*}W_{3:3}^{*}W_{3:4}^{*}W_{3:5}^{*}W_{3:6}^{*}W_{3:7}^{*}\\ W_{4;1}^{*}W_{4:2}^{*}W_{4:3}^{*}W_{4:4}^{*}W_{4:5}^{*}W_{4:6}^{*}W_{4:7}^{*}\\ W_{5:1}^{*}W_{5:2}^{*}W_{5:3}^{*}W_{5:4}^{*}W_{5:5}^{*}W_{5:6}^{*}W_{5:7}^{*}\\ W_{6:1}^{*}W_{6:2}^{*}W_{6:3}^{*}W_{6:4}^{*}W_{6:5}^{*}W_{6:6}^{*}W_{6:7}^{*}\\ W_{7:1}^{*}W_{7:2}^{*}W_{7:3}^{*}W_{7:4}^{*}W_{7:5}^{*}W_{7:6}^{*}W_{7:7} \end{array}\right]$$

The units $\left\{W_{1\left(2\right)}^{*},W_{2\left(6\right)}^{*},W_{3\left(4\right)}^{*},W_{4\left(1\right)}^{*},W_{5\left(1\right)}^{*},W_{6\left(7\right)}^{*},W_{7\left(7\right)}^{*}\right\}$ in boxes represent MMQDRSS of size n=7.

### 1.7 Estimation of the population mean and variance

To compute the sample, mean, and variance of the MMQDRSS, four cases are discussed.

1. When m is an even number, c is an even number, and m-c is an even number.

$${\bar{W}}_{\left(MMQDRSS\right)}^{*}=\frac{1}{mr}\sum_{j=1}^{r}\left(\sum_{i=1}^{\frac{c}{2}}W_{i\left(\frac{m+1}{4}\right)}^{*}+\sum_{i=\frac{c}{2}+1}^{c}W_{i\left(3\left(\frac{m+1}{4}\right)\right)}^{*}+\sum_{i=c+1}^{\frac{m-c}{2}}W_{i\left(\;1,m\right)}^{*}+\sum_{i=\frac{m-c}{2}+1}^{m}W_{i\left(m,m\right)}^{*}\right)$$

And variance,

$$Var\left({\bar{W}}_{\left(MMQDRSS\right)}^{*}\right)=\frac{1}{m^{2}\;r}\left(\frac{c}{2}\;Var\;\left(Z_{i\left(\frac{m+1}{4}\right)}^{*}\right)+\frac{c}{2}Var\left(Z_{i\left(3\left(\frac{m+1}{4}\right)\right)}^{*}\right)+\frac{m-c}{2}Var\left(Z_{i\left(\;1,m\right)}^{*}\right)+\frac{m-c}{2}Var\left(Z_{i\left(m,m\right)}^{*}\right)\right)$$

2. When m is an even number, c is an odd number, and m-c is an odd number.

$${\bar{W}}_{\left(MMQDRSS\right)}^{*}=\frac{1}{mr}\sum_{j=1}^{r}\left(\sum_{i=1}^{\frac{c-1}{2}}W_{i\left(\frac{m+1}{4}\right)}^{*}+\sum_{i=\frac{c-1}{2}+1}^{c}W_{i\left(3\left(\frac{m+1}{4}\right)\right)}^{*}+W_{i\left(\frac{m+1}{2}\right)}^{*}+\sum_{i=c+1}^{\frac{m-c-1}{2}}W_{i\left(\;1,m\right)}^{*}+\sum_{i=\frac{m-c-1}{2}+1}^{m-1}W_{i\left(m,m\right)}^{*}+W_{m\left(1,m\right)}^{*}\right)$$

And variance,

$$Var\left({\bar{W}}_{\left(MMQDRSS\right)}^{*}\right)=\frac{1}{m^{2}\;r}\left(\frac{c-1}{2}\;Var\;\left(W_{i\left(\frac{m+1}{4}\right)}^{*}\right)+\frac{c-1}{2}Var\left(W_{i\left(3\left(\frac{m+1}{4}\right)\right)}^{*}\right)+Var\left(W_{i\left(\frac{m+1}{2}\right)}^{*}\right)+\frac{m-c-1}{2}Var\left(W_{i\left(\;1,m\right)}^{*}\right)+\frac{m-c-1}{2}Var\left(W_{i\left(m,m\right)}^{*}\right)+Var\left(W_{m\left(1,m\right)}^{*}\right)\right)$$

3. When both m and c are odd, m-c is even.

$${\bar{W}}_{\left(MMQDRSS\right)}^{*}=\frac{1}{mr}\sum_{j=1}^{r}\left(\sum_{i=1}^{\frac{c-1}{2}}W_{i\left(\frac{m+1}{4}\right)}^{*}+\sum_{i=\frac{c-1}{2}+1}^{c}W_{i\left(3\left(\frac{m+1}{4}\right)\right)}^{*}+W_{i\left(\frac{m+1}{2}\right)}^{*}+\sum_{i=c+1}^{\frac{m-c-1}{2}}W_{i\left(\;1,m\right)}^{*}+\sum_{i=\frac{m-c-1}{2}+1}^{m-1}W_{i\left(m,m\right)}^{*}\right)$$

And variance,

$$Var\left({\bar{W}}_{\left(MMQDRSS\right)}^{*}\right)=\frac{1}{m^{2}\;r}\left(\frac{c-1}{2}\;Var\;\left(W_{i\left(\frac{m+1}{4}\right)}^{*}\right)+\frac{c-1}{2}Var\left(W_{i\left(3\left(\frac{m+1}{4}\right)\right)}^{*}\right)+Var\left(W_{i\left(\frac{m+1}{2}\right)}^{*}\right)+\frac{m-c}{2}Var\left(W_{i\left(\;1,m\right)}^{*}\right)+\frac{m-c}{2}Var\left(W_{i\left(m,m\right)}^{*}\right)\right)$$

4. When m is an odd number, c is an even number, and m-c is an odd number.

$${\bar{W}}_{\left(MMQDRSS\right)}^{*}=\frac{1}{mr}\sum_{j=1}^{r}\left(\sum_{i=1}^{\frac{c}{2}}W_{i\left(\frac{m+1}{4}\right)}^{*}+\sum_{i=\frac{c}{2}+1}^{c}W_{i\left(3\left(\frac{m+1}{4}\right)\right)}^{*}+\sum_{i=c+1}^{\frac{m-c-1}{2}}W_{i\left(\;1,m\right)}^{*}+\sum_{i=\frac{m-c-1}{2}+1}^{m-1}W_{i\left(m,m\right)}^{*}+W_{m\left(1,m\right)}^{*}\right)$$

And variance,

$$Var\left({\bar{W}}_{\left(MMQDRSS\right)}^{*}\right)=\frac{1}{m^{2}\;r}\left(\frac{c}{2}\;Var\;\left(W_{i\left(\frac{m+1}{4}\right)}^{*}\right)+\frac{c}{2}Var\left(W_{i\left(3\left(\frac{m+1}{4}\right)\right)}^{*}\right)+\frac{m-c-1}{2}Var\left(W_{i\left(\;1,m\right)}^{*}\right)+\frac{m-c-1}{2}Var\left(W_{i\left(m,m\right)}^{*}\right)+Var\left(W_{m\left(1,m\right)}^{*}\right)\right)$$

These estimators are unbiased (See Appendix).

### 1.8 Simulation study

To evaluate the performance of our method, we will use the Yasin [17] simulation scheme, which is as follows: We compare the performance of the MMQDRSS mean estimator to other estimators in DRSS, EDRSS, MDRSS, and QDRSS across various probability distributions, both symmetrical and asymmetrical, in a simulation study. These include the normal (0,1), uniform (0,1), lognormal (0,1), Weibull (6), exponential (1), gamma (4), gamma (2,1), beta (7,4), logistic (0,1), chi-sq (1), student T (3), weibull (6,1), and Cauchy (0,1) distributions. There are 100,000 runs of a Monte Carlo simulation in R-Language to see how well the DRSS, EDRSS, MDRSS, and QDRSS work compared to chance-picking. In Table 1, we look at how well the mean estimators work for one cycle with m = 5,6.

$$Eff\left({\bar{W}}_{MMQDRSS\;},{\bar{W}}_{SRS\;}\right)=\frac{Var\left({\bar{W}}_{SRS\;}\right)}{Var\left({\bar{W}}_{MMQDRSS\;}\right)}\times 100$$

**Table 1:** Efficiency of MMQDRSS, QDRSS, MDRSS and DRSS (m = 5 and m = 6).

Distributions	m = 5					m = 6				
	DRSS	EDRSS	MDRSS	QDRSS	MMQDRSS	DRSS	EDRSS	MDRSS	QDRSS	MMQDRSS
Normal (0,1)	4.46	3.44	7.33	6.41	12.54	5.57	3.39	9.43	7.34	13.65
Lognormal (0,1)	1.88	0.99	27.54	14.28	10.83	1.98	0.84	32.66	11.30	12.53
Weibull (6)	4.46	3.46	7.23	6.29	12.30	5.47	3.44	9.34	7.17	13.39
Exponential (1)	3.04	1.90	10.69	7.57	17.48	3.52	1.66	13.15	7.24	18.18
Gamma (4)	3.97	2.80	8.01	6.70	12.57	4.71	2.65	9.94	7.19	14.53
Gamma (2,1)	3.59	2.40	8.82	7.00	13.51	4.10	2.15	10.82	7.14	15.46
Beta (7,4)	4.69	3.89	6.65	5.99	41.45	5.89	3.95	8.52	6.94	42.43
Logistic (0,1)	3.82	2.57	9.24	7.39	15.21	4.64	2.37	11.55	7.89	16.59
Chi-sq (1)	2.44	1.43	15.90	8.72	24.92	2.81	1.19	18.86	7.23	24.47
Student T (3)	2.13	1.23	16.87	12.12	26.96	2.55	0.92	21.53	11.97	30.70
Weibull (6,1)	4.48	3.48	7.36	6.35	12.42	5.50	3.43	9.26	7.17	13.57

**Fig. 1:**
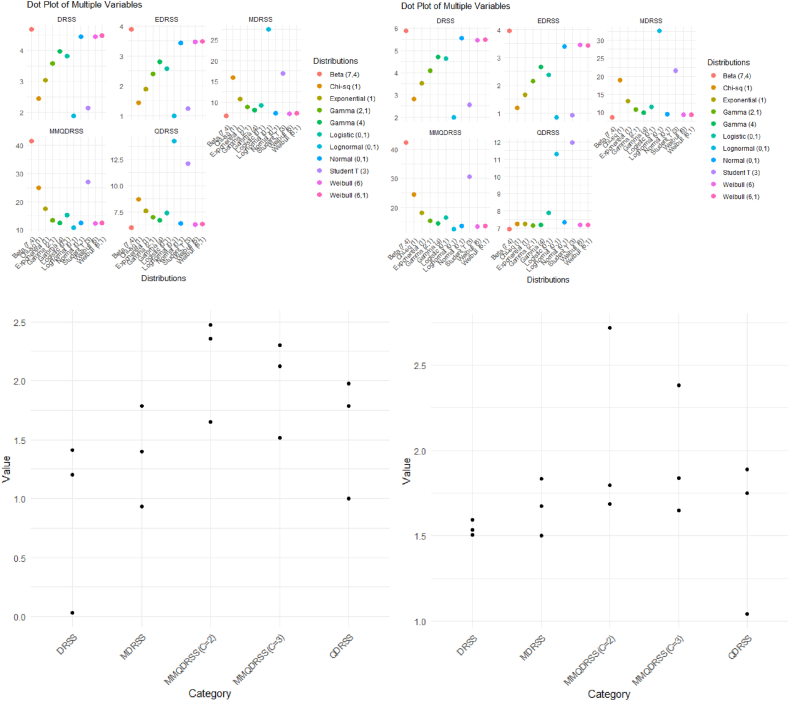
Relative efficiency of the real-life data set.

In relation to efficiency (Eff), the proposed modified median double-ranked set sampling (MMQDRSS) considers the best plan (scheme) for DRSS, EDRSS, MDRSS, QDRSS, MMQDRSS, and SRS cases for all schemes. The simulated results show that the efficacy of the proposed ${\bar{W}}_{\left(MMQDRSS\right)}$ is an increasing function of m. It is remarkable (and interesting) to note that the proposed ${\bar{W}}_{\left(MMQDRSS\right)}$ performance is efficient from the ${\bar{W}}_{\left(DRSS\right)}\text{,}$ ${\bar{W}}_{\left(EDRSS\;\right)}$, ${\bar{W}}_{\left(MDRSS\right)}$, ${\bar{W}}_{\left(QDRSS\right)}$ and ${\bar{W}}_{\left(SRS\right)}$ in both symmetrical and non-symmetrical populations. Under the studied distribution, there is a significant variance in the efficacy of the population mean estimator applying MMQDRSS versus alternative methods. The best results are obtained from the Beta (7,4) population. The efficiency plot is a valuable tool in performance evaluation for various statistical metrics such as DRSS, EDRSS, MDRSS, QDRSS, and MMQDRSS within the context of simulation studies. For (m = 5, m = 6). In DRSS, Weibull (6,1) gives the efficient result of efficiency. EDRSS gives the best results with beta (7, 4). In MDRSS and QDRSS, log normal (0, 1) gives higher efficiency. Hence, Beta (7,4) gives the highest efficiency in our proposed method, MMQDRSS Fig. 1.

## 2 Real life data sets

In the following part of this article, we will discuss the precise data sets used in this work. We will discuss the origins, composition, and pre-use verification procedures of the objects in question. The Hong Kong Children Data from 1993 obtained from the Growth Survey, the U.S. Census of Agriculture Data from 1992, and data collected by Rita Gnap in 1995 were all used with authorized consent [1]. Determine and contrast the relative efficacy of QDRSS, MDRSS, and DRSS with that of the suggested approach, MMQDRSS. Table 2 shows that MMQDRSS outperforms QDRSS, MDRSS, and DRSS in terms of relative efficiency for c = 2 and c = 3. Fig. 1 shows the relative efficiency of the real-life data set.

$$R.Eff\left({\bar{W}}_{MMQDRSS\;},{\bar{W}}_{SRS\;}\right)=\frac{Var\left({\bar{W}}_{SRS\;}\right)}{Var\left({\bar{W}}_{MMQDRSS\;}\right)}$$

**Table 2:** Relative efficiency for real life data sets.

m = 5	Hong Kong Children Data 1993 by Growth Survey	Census of Agriculture Data from the U.S. 1992	Data courtesy of Rita Gnap 1995
MMQDRSS(C = 2)	1.653	2.354	2.475
MMQDRSS(C = 3)	1.516	2.122	2.302
QDRSS	0.998	1.784	1.978
MDRSS	0.931	1.785	1.399
DRSS	1.412	1.201	0.028
m = 6			
MMQDRSS(C = 2)	1.687	2.72	1.795
MMQDRSS(C = 3)	1.649	2.38	1.837
QDRSS	1.041	1.891	1.75
MDRSS	1.502	1.836	1.673
DRSS	1.536	1.595	1.506

## 3 Conclusion and discussion

The study looked at how well the suggested modified median double-ranked set sampling (MMQDRSS) worked compared to other sampling methods like DRSS, EDRSS, MDRSS, QDRSS, and SRS in a number of different situations. The results consistently demonstrate that MMQDRSS outperforms these schemes in terms of efficiency. Notably, the efficiency of MMQDRSS, represented as ${\bar{W}}_{\left(MMQDRSS\right)}$, increases with the sample size (m). Importantly, MMQDRSS proves to be more efficient than DRSS, EDRSS, MDRSS, QDRSS, and SRS in both symmetrical and non-symmetrical populations. The difference between how well MMQDRSS estimates the population mean compared to other methods is very big, with the Beta (7,4) population showing the best results.

In this article, we discussed the efficiency of the MMQDRSS sampling method in comparison to other established schemes. The data sets used in the study, including Hong Kong Children Data 1993 from the Growth Survey, Census of Agriculture Data from the U.S. 1992, and data courtesy of Rita Gnap 1995, were introduced, and their sources, characteristics, and preprocessing steps for data quality assurance were outlined. The primary focus of the study was to compute and compare the relative efficiency of MMQDRSS against QDRSS, MDRSS, and DRSS. The results consistently favored MMQDRSS, with Table data showing that MMQDRSS (for both c = 2 and c = 3) consistently outperforms QDRSS, MDRSS, and DRSS across various scenarios. This underscores the superiority of MMQDRSS as an effective sampling scheme for population mean estimation in different population distributions. The most important thing when using the MMQDRSS method is choosing the right sample size. We consider all populations and rank them, and then, after ranking, we select samples from M sets. The selection of samples in M sets is contingent upon the size of the population. There are several useful ways to rank samples, such as pairwise ranking and point allocation, which help choose the right samples. One of its drawbacks is the rarity of a single sample. It's possible that an element didn't make the cut, but this is an uncommon occurrence. On a regular basis, MMQDRSS uses datasets from various situations to see if the suggested method is still useful in the real world.

In conclusion, the MMQDRSS method is a better and more accurate estimator than other methods in both symmetrical and non-symmetrical groups. The choice of distribution and the value of m have a big impact on how well the estimator works. In our simulations, Beta (7,4) regularly showed impressive efficiency. It's clear from these results that MMQDRSS is an important part of real statistical sampling and estimates. The in-depth study of many real-world datasets repeatedly shows that MMQDRSS is better at reducing variance and working efficiently. MMQDRSS always does better than tried-and-true methods, which makes it useful in a wide range of sampling situations. In fields like education, agriculture, and population data, where accurate estimates are needed to make smart decisions, it could be used.

## CRediT authorship contribution statement

**Muhammad Ahmed Shehzad:** Writing – review & editing, Validation, Supervision, Software, Project administration, Investigation, Funding acquisition, Formal analysis, Conceptualization. **Anam Nisar:** Writing – original draft, Visualization, Software, Methodology, Investigation, Formal analysis, Data curation. **Aamna Khan:** Resources, Methodology, Formal analysis, Conceptualization. **Walid Emam:** Writing – review & editing, Resources, Investigation, Funding acquisition, Data curation. **Yusra Tashkandy:** Validation, Resources, Methodology, Funding acquisition, Formal analysis. **Haris Khurram:** Visualization, Validation, Investigation, Formal analysis. **Isra Al-Shbeil:** Visualization, Resources, Project administration, Methodology, Data curation.

## Declaration of competing interest

The authors declare that they have no known competing financial interests or personal relationships that could have appeared to influence the work reported in this paper.

## Appendix

### Estimation of population mean and variance

To compute the sample, mean, and variance of the MMQDRSS, four cases are discussed.

1. When m is an even number, c is an even number, and m-c is an even number.
2. When m is an even number, c is an odd number, and m-c is an odd number.
3. When both m and c are odd, m-c is even.
4. When m is an odd number, c is an even number, and m-c is an odd number.
1. When m is an even number, c is an even number, and m-c is an even number

**Example:**

For *m* = 4, *c* = 2, and m-c = 2 the MMRSS can be selected as follows.

To select an MMQDRSS of size n=4 for r=1(m=4,c=2), identify $m^{3}=64$ (4 sets of 16 sampling units each). Consider, $Z_{i\left(j\right)k}$ become $j^{th}$ the lowest ranked unit from $i^{th}$ subsection of the set $k^{th}$, in which i,j,k=1,…,4. Order the units in each subset of the five sets based on the variable being studied.

(I) $$\begin{array}{c} \begin{array}{c} \left[\begin{array}{c} W_{11\left(1\right)}W_{12\left(1\right)}W_{13\left(1\right)}W_{14\left(1\right)}\\ W_{21\left(1\right)}W_{22\left(1\right)}W_{23\left(1\right)}W_{24\left(1\right)}\\ {\;W}_{31\left(1\right)}W_{32\left(1\right)}{\;W}_{33\left(1\right)}W_{34\left(1\right)}\\ W_{41\left(1\right)}W_{42\left(1\right)}W_{43\left(1\right)}W_{44\left(1\right)} \end{array}\right]\\ \left[\begin{array}{c} W_{11\left(2\right)}W_{12\left(2\right)}W_{13\left(2\right)}W_{14\left(2\right)}\\ W_{21\left(2\right)}W_{22\left(2\right)}W_{23\left(2\right)}W_{24\left(2\right)}\\ W_{31\left(2\right)}W_{32\left(2\right)}W_{33\left(2\right)}W_{34\left(2\right)}\\ W_{41\left(2\right)}W_{42\left(2\right)}W_{43\left(2\right)}W_{44\left(2\right)} \end{array}\right]\\ \vdots \end{array}\\ \left[\begin{array}{c} W_{11\left(4\right)}W_{12\left(4\right)}W_{13\left(4\right)}W_{14\left(4\right)}\\ W_{21\left(4\right)}W_{22\left(4\right)}W_{23\left(4\right)}W_{24\left(4\right)}\\ W_{31\left(4\right)}W_{32\left(4\right)}W_{33\left(4\right)}W_{34\left(4\right)}\\ W_{41\left(4\right)}W_{42\left(4\right)}W_{43\left(4\right)}W_{44\left(4\right)} \end{array}\right] \end{array}$$

Then, from set c, select the middle units in the boxes and get average of these middle units, each set's sampling units are listed in rows from eq(I), as follows:

$$\left[\begin{array}{c} W_{11\left(1\right)}W_{12\left(1\right)}W_{13\left(1\right)}W_{14\left(1\right)}\\ W_{21\left(2\right)}W_{22\left(2\right)}W_{23\left(2\right)}W_{24\left(2\right)}\\ W_{31\left(3\right)}W_{32\left(3\right)}W_{33\left(3\right)}W_{34\left(3\right)}\\ W_{41\left(4\right)}W_{42\left(4\right)}W_{43\left(4\right)}W_{44\left(4\right)} \end{array}\right]$$

With no determining the real measurement of the sub-section units, rank the units each subsection, the preceding once more set. Then, select ${\left(\frac{m+1}{4}\right)}^{th}$ a ranked unit (in boxes), $W_{i\left(1:2\right)}^{*}$ to the $i^{th}$ sub-section (i=1,2) and select extremes unit of rank (in boxes), i.e., $W_{i\left(3:4\right)}^{*}$ to $i^{th}$ sub-section (i=3,4) actual estimation is listed below:

$$\left[\begin{array}{c} {W_{1:1}^{*}W}_{1:2}^{*}W_{1:3}^{*}W_{1:4}^{*}\\ W_{2:1}^{*}W_{2:2}^{*}W_{2:3}^{*}W_{2:4}^{*}\\ W_{3:1}^{*}W_{3:2}^{*}W_{3:3}^{*}W_{3:4}^{*}\\ W_{4:1}^{*}W_{4:2}^{*}W_{4:3}^{*}W_{4:4}^{*} \end{array}\right]$$

The units $\left\{W_{1:1\;}^{*},W_{2:4}^{*},W_{3:1}^{*},W_{4:4}^{*}\right\}$ in boxes represent MMQDRSS of size n=4. The mean of these four sampling units is defined population mean estimator as:

$${\bar{W}}_{\left(MMQDRSS\right)}^{*}=\frac{W_{1:1\;}^{*}+W_{2:4}^{*}+W_{3:1}^{*}+W_{4:4}^{*}}{4}$$

Consider the point that, $W_{1},W_{2},\ldots ,W_{n}$ be an n random sample using a density function distribution $f_{W}$, $function\;of\;distribution\;F_{W}$, the mean is μ and the variance is $\sigma^{2}$. The SRS indicates mean is that ${\bar{W}}_{SRS}=\sum_{i=1}^{n}W_{i}/n$ and $E\left({\bar{W}}_{SRS}\right)=\mu$ along with $Var\left({\bar{W}}_{SRS}\right)=\sigma^{2}/n$. In this research, the cycle is repeated once, i.e., r=1. Consider $W_{i\left(\frac{m+1}{4}\right)}^{*}$ displays the unit from the firstquartile the $i^{th}$ sub-section $\left(i=1,\ldots ,\frac{c}{2}\right)$ , $W_{i\;\left(3\left(\frac{m+1}{4}\right)\right)}^{*}$ displays the third quartile unit from the $i^{th}$ sub-section $\left(i=\frac{c}{2}+1,\ldots ,c\right)$, $W_{i\left(1,m\right)}^{*}$ and $W_{i\left(m,m\right)}^{*}$ shows the selected unites of extreme rank set sampling (even) from the $i^{th}$ subset of m−c.

The purposed estimator as:

(1) $${\bar{W}}_{\left(MMQDRSS\right)}^{*}=\frac{1}{mr}\sum_{j=1}^{r}\left(\sum_{i=1}^{\frac{c}{2}}W_{i\left(\frac{m+1}{4}\right)}^{*}+\sum_{i=\frac{c}{2}+1}^{c}W_{i\left(3\left(\frac{m+1}{4}\right)\right)}^{*}+\sum_{i=c+1}^{\frac{m-c}{2}}W_{i\left(\;1,m\right)}^{*}+\sum_{i=\frac{m-c}{2}+1}^{m}W_{i\left(m,m\right)}^{*}\right)$$

#### Theorem 1.1

${\bar{W}}_{\left(MMQDRSS\right)}^{*}$ is an unbiased population mean estimator.

**Proof:**

Let m is even, apply expectation on both sides of eq (1),

$$E\left({\bar{W}}_{\left(MMQDRSS\right)}^{*}\right)=E\left[\frac{1}{mr}\sum_{j=1}^{r}\left(\sum_{i=1}^{\frac{c}{2}}W_{i\left(\frac{m+1}{4}\right)}^{*}+\sum_{i=\frac{c}{2}+1}^{c}W_{i\left(3\left(\frac{m+1}{4}\right)\right)}^{*}+\sum_{i=c+1}^{\frac{m-c}{2}}W_{i\left(\;1,m\right)}^{*}+\sum_{i=\frac{m-c}{2}+1}^{m}W_{i\left(m,m\right)}^{*}\right)\right]$$

$$E\left({\bar{W}}_{\left(MMQDRSS\right)}^{*}\right)=\frac{1}{mr}\;r\;\left(\frac{c}{2}\;E\;\left(W_{i\left(\frac{m+1}{4}\right)}^{*}\right)+\frac{c}{2}E\left(W_{i\left(3\left(\frac{m+1}{4}\right)\right)}^{*}\right)+\frac{m-c}{2}E\left(w_{i\left(\;1,m\right)}^{*}\right)+\frac{m-c}{2}E\left(W_{i\left(m,m\right)}^{*}\right)\right)$$

Where $E\;\left(W_{i\left(\frac{m+1}{4}\right)}^{*}\right)=\mu_{i\left(\frac{m+1}{4}\right)}^{*}$, $E\left(W_{i\left(3\left(\frac{m+1}{4}\right)\right)}^{*}\right)=\mu_{i\left(3\left(\frac{m+1}{4}\right)\right)}^{*}$, $E\left(W_{i\left(\;1,m\right)}^{*}\right)=\mu_{i\left(\;1,m\right)}^{*}$. and $E\left(W_{i\left(m,m\right)}^{*}\right)=\mu_{i\left(m,m\right)}^{*}$. Using symmetric condition with regard to μ by David and Nagaraja (2004), it has ${{{\mu^{*}}_{i\left(\frac{m+1}{4}\right)}=\mu }^{*}}_{i\left(3\left(\frac{m+1}{4}\right)\right)}=\mu_{i\left(\;1,m\right)}^{*}=\mu_{i\left(m,m\right)}^{*}=\mu^{*}$ and also $\mu^{*}=\mu$. Therefore,

$$E\left({\bar{w}}_{\left(MMQDRSS\right)}^{*}\right)=\frac{1}{mr}\;r\;\left(\frac{c}{2}\mu +\frac{c}{2}\mu +\frac{m-c}{2}\mu +\frac{m-c}{2}\mu \right)$$

$$E\left({\bar{W}}_{\left(MMQDRSS\right)}^{*}\right)=\mu$$

If m is even then the variance of ${\bar{W}}_{\left(MMQDRSS\right)}^{*}$ by using eq (2) is,

$$Var\left({\bar{W}}_{\left(MMQDRSS\right)}^{*}\right)=Var\left[\frac{1}{mr}\sum_{j=1}^{r}\left(\sum_{i=1}^{\frac{c}{2}}W_{i\left(\frac{m+1}{4}\right)}^{*}+\sum_{i=\frac{c}{2}+1}^{c}W_{i\left(3\left(\frac{m+1}{4}\right)\right)}^{*}+\sum_{i=c+1}^{\frac{m-c}{2}}W_{i\left(\;1,m\right)}^{*}+\sum_{i=\frac{m-c}{2}+1}^{m}{W\;}_{i\left(m,m\right)}^{*}\right)\right]$$

(2) $$Var\left({\bar{W}}_{\left(MMQDRSS\right)}^{*}\right)=\frac{1}{m^{2}\;r}\left(\frac{c}{2}\;Var\;\left(Z_{i\left(\frac{m+1}{4}\right)}^{*}\right)+\frac{c}{2}Var\left(Z_{i\left(3\left(\frac{m+1}{4}\right)\right)}^{*}\right)+\frac{m-c}{2}Var\left(Z_{i\left(\;1,m\right)}^{*}\right)+\frac{m-c}{2}Var\left(Z_{i\left(m,m\right)}^{*}\right)\right)$$

2. When m is an even number, c is an odd number, and m-c is an odd number

**Example**

For m=6, c=3, and m−c=3, the MMQDRSS can be selected as follows.

To select an MMQDRSS in size n=6 have been taken for r=1(m=3,c=3), detect $m^{3}=216$ (6 sets of 36 sampling units each). Consider, $Z_{i\left(j\right)k}$ become $j^{th}$ the lowest ranked unit from $i^{th}$ subsection of the set $k^{th}$, in which i,j,k=1,2,3,…,5,6. Order the units in each subset of the five sets based on the variable being studied.

(J) $$\begin{array}{c} \begin{array}{c} \left[\begin{array}{c} W_{11\left(1\right)}W_{12\left(1\right)}W_{13\left(1\right)}W_{14\left(1\right)}W_{15\left(1\right)}W_{16\left(1\right)}\\ W_{21\left(1\right)}W_{22\left(1\right)}W_{23\left(1\right)}W_{24\left(1\right)}W_{25\left(1\right)}W_{26\left(1\right)}\\ W_{31\left(1\right)}W_{32\left(1\right)}W_{33\left(1\right)}W_{34\left(1\right)}W_{35\left(1\right)}W_{36\left(1\right)}\\ {W_{41\left(1\right)}\;W}_{42\left(1\right)}W_{43\left(1\right)}W_{44\left(1\right)}W_{45\left(1\right)}W_{46\left(1\right)}\\ W_{51\left(1\right)}W_{52\left(1\right)\;W_{53\left(1\right)}}W_{54\left(1\right)}W_{55\left(1\right)}W_{56\left(1\right)}\\ W_{61\left(1\right)}W_{62\left(1\right)}W_{63\left(1\right)}W_{64\left(1\right)}W_{65\left(1\right)\;}W_{66\left(1\right)} \end{array}\right]\\ \left[\begin{array}{c} W_{11\left(2\right)}W_{12\left(2\right)}W_{13\left(2\right)\;}W_{14\left(2\right)}W_{15\left(2\right)}W_{16\left(2\right)}\\ W_{21\left(2\right)}W_{22\left(2\right)}W_{23\left(2\right)}W_{24\left(2\right)}\;W_{25\left(2\right)}\;W_{26\left(2\right)}\\ W_{31\left(2\right)}W_{32\left(2\right)}W_{33\left(2\right)}W_{34\left(2\right)}W_{35\left(2\right)}W_{36\left(2\right)}\\ {W_{41\left(2\right)}\;W}_{42\left(2\right)}W_{43\left(2\right)}W_{44\left(2\right)}W_{45\left(2\right)}W_{46\left(2\right)}\\ W_{51\left(2\right)}W_{52\left(2\right)}W_{53\left(2\right)}W_{54\left(2\right)}W_{55\left(2\right)}W_{56\left(2\right)}\\ W_{61\left(2\right)}W_{62\left(2\right)}W_{63\left(2\right)}W_{64\left(2\right)}W_{65\left(2\right)\;}W_{66\left(2\right)} \end{array}\right]\\ \vdots \end{array}\\ \left[\begin{array}{c} W_{11\left(6\right)}W_{12\left(6\right)}W_{13\left(6\right)\;}W_{14\left(6\right)}W_{15\left(6\right)}W_{16\left(6\right)}\\ W_{21\left(6\right)}W_{22\left(6\right)}W_{23\left(6\right)}W_{24\left(6\right)}W_{25\left(6\right)}W_{26\left(6\right)}\\ W_{31\left(6\right)}W_{32\left(6\right)}W_{33\left(6\right)}W_{34\left(6\right)}W_{35\left(6\right)}W_{36\left(6\right)}\\ {W_{41\left(6\right)}\;W}_{42\left(6\right)}W_{43\left(6\right)}W_{44\left(6\right)}W_{45\left(6\right)}W_{46\left(6\right)}\\ W_{51\left(2\right)}W_{52\left(2\right)}W_{53\left(6\right)}W_{54\left(6\right)}{WW}_{55\left(6\right)}W_{56\left(6\right)}\\ W_{61\left(2\right)}W_{62\left(2\right)}W_{63\left(6\right)}W_{64\left(6\right)}W_{65\left(6\right)\;}W_{66\left(6\right)} \end{array}\right] \end{array}$$

Then, from set c, select the middle units in the boxes and get average of these middle units, each set of sampling units are listed in rows from eg (J), as follows:

$$\left[\begin{array}{c} W_{11\left(1\right)}W_{12\left(1\right)}W_{13\left(1\right)}W_{14\left(1\right)}W_{15\left(1\right)}W_{16\left(1\right)}\\ W_{21\left(2\right)}W_{22\left(2\right)}W_{23\left(2\right)}W_{24\left(2\right)}W_{25\left(2\right)}W_{26\left(2\right)}\\ W_{31\left(3\right)}W_{32\left(3\right)}W_{3\left(3\right)}W_{34\left(3\right)}W_{35\left(3\right)}W_{36\left(3\right)}\\ W_{41\left(4\right)}W_{42\left(4\right)}W_{43\left(4\right)}W_{44\left(4\right)}W_{45\left(4\right)}W_{46\left(4\right)}\\ W_{51\left(5\right)}W_{52\left(5\right)}W_{53\left(5\right)}W_{54\left(5\right)}W_{55\left(5\right)}W_{56\left(5\right)}\\ W_{61\left(6\right)}W_{62\left(6\right)}W_{63\left(6\right)}W_{64\left(6\right)}W_{65\left(6\right)}W_{66\left(6\right)} \end{array}\right]$$

With no determining the real measurement these sub-sections units, sort the unit of each subsection the preceding set once more. Then, select ${\left(\frac{m+1}{4}\right)}^{th}$ a sorting unit (in blocks), $W_{i\left(1:3\right)}^{*}$ to the $i^{th}$ sub-section (i=1,2,3) and select extremes unit of rank (in blocks), i.e., $W_{i\left(4:6\right)}^{*}$ to $i^{th}$ sub-section (i=4,5,6) actual estimation is listed below:

$$\left[\begin{array}{c} W_{1:1}^{*}\;W_{1:2}^{*}\;W_{1:3}^{*}\;W_{1:4}^{*}\;W_{1:5}^{*}W_{1:6}^{*}\\ W_{2:1}^{*}W_{2:2}^{*}\;W_{2:3}^{*}W_{2:4}^{*}\;W_{2:5}^{*}\;W_{2:6}^{*}\\ W_{3:1}^{*}W_{3:2}^{*}W_{3:3}^{*}W_{3:4}^{*}{\;W}_{3:5}^{*}{\;W}_{3:6}^{*}\\ W_{4:1}^{*}{\;W}_{4:2\;}^{*}W_{4:3}^{*}\;W_{4:4}^{*}{\;W}_{4:5}W_{4:6}^{*}\\ W_{5:1}^{*}W_{5:2}^{*}W_{5:3}^{*}W_{5:4}^{*}W_{5:5}\;W_{5:6}^{*}\\ W_{6:1}^{*}W_{6:2}^{*}W_{6:3}^{*}W_{6:4}^{*}W_{6:5}^{*}W_{6:6}^{*} \end{array}\right]$$

The above specified units $\left\{W_{1\left(2\right)}^{*},W_{2\left(5\right)}^{*},\frac{1}{2}\left(W_{3\left(3\right)}^{*}+W_{3\left(4\right)}^{*}\right),W_{4\left(1\right)}^{*},W_{5\left(6\right)}^{*},\frac{1}{2}\left(W_{6\left(1\right)}^{*}+W_{6\left(6\right)}^{*}\right)\right\}$ in boxes represent MMQDRSS of size n=6. The population mean estimator is defined as the mean of these four sampling units:

$${\bar{W}}_{\left(MMQDRSS\right)}^{*}=\frac{\left\{W_{1\left(2\right)}^{*}+W_{2\left(5\right)}^{*}+\frac{1}{2}\left(W_{3\left(3\right)}^{*}+W_{3\left(4\right)}^{*}\right)+W_{4\left(1\right)}^{*}+W_{5\left(6\right)}^{*}+\frac{1}{2}\left(W_{6\left(1\right)}^{*}+W_{6\left(6\right)}^{*}\right)\right\}}{6}$$

Consider the point that, $W_{1},W_{2},\ldots ,W_{n}$ be an n random sample using a density function distribution a density function $f_{W}$, function of distribution $F_{W}\text{,}$ the mean is μ,t he variance is $\sigma^{2}$. The SRS indicates mean is that, ${\bar{W}}_{SRS}=\sum_{i=1}^{n}W_{i}/n$ and $E\left({\bar{W}}_{SRS}\right)=\mu$ along with $Var\left({\bar{W}}_{SRS}\right)=\sigma^{2}/n$. In this research, the cycle is repeated once, i.e., r=1. Consider $W_{i\left(\frac{m+1}{4}\right)}^{*}$ displays first quartile unit taken $i^{th}$ sub-section $\left(i=1,2,\ldots ,\frac{c}{2}\right)$, $W_{i\;\left(3\left(\frac{m+1}{4}\right)\right)}^{*}$ displays the third quartile unit the $i^{th}$ sub-section $\left(i=\frac{c-1}{2}+1,\ldots ,c\right)$, $W_{i\left(\frac{m+1}{2}\right)}^{*}$ shows the median unit from the $i^{th}$ subset of $\left(i=c\right),W_{i\left(\;1,m\right)}^{*}$, $W_{i\left(m,m\right)}^{*}$ and $W_{m\left(1,m\right)}^{*}$ shows the selected unites of extreme rank set sampling (odd) from the $i^{th}$ subset of m−c.

The purposed estimator as:

(3) $${\bar{W}}_{\left(MMQDRSS\right)}^{*}=\frac{1}{mr}\sum_{j=1}^{r}\left(\sum_{i=1}^{\frac{c-1}{2}}W_{i\left(\frac{m+1}{4}\right)}^{*}+\sum_{i=\frac{c-1}{2}+1}^{c}W_{i\left(3\left(\frac{m+1}{4}\right)\right)}^{*}+W_{i\left(\frac{m+1}{2}\right)}^{*}+\sum_{i=c+1}^{\frac{m-c-1}{2}}W_{i\left(\;1,m\right)}^{*}+\sum_{i=\frac{m-c-1}{2}+1}^{m-1}W_{i\left(m,m\right)}^{*}+W_{m\left(1,m\right)}^{*}\right)$$

#### 2.1. Theorem

${\bar{W}}_{\left(MMQDRSS\right)}^{*}$ is an unbiased population mean estimator.

**Proof:**

Let m is an even, apply expectation on both sides of eq (3),

$$E\left({\bar{W}}_{\left(MMQDRSS\right)}^{*}\right)=E\left[\frac{1}{mr}\sum_{j=1}^{r}\left(\sum_{i=1}^{\frac{c-1}{2}}W_{i\left(\frac{m+1}{4}\right)}^{*}+\sum_{i=\frac{c-1}{2}+1}^{c}W_{i\left(3\left(\frac{m+1}{4}\right)\right)}^{*}+W_{i\left(\frac{m+1}{2}\right)}^{*}+\sum_{i=c+1}^{\frac{m-c-1}{2}}W_{i\left(\;1,m\right)}^{*}+\sum_{i=\frac{m-c-1}{2}+1}^{m-1}W_{i\left(m,m\right)}^{*}+W_{m\left(1,m\right)}^{*}\right)\right]$$

$$E\left({\bar{W}}_{\left(MMQDRSS\right)}^{*}\right)=\frac{1}{mr}\;r\;\left(\frac{c-1}{2}\;E\;\left(W_{i\left(\frac{m+1}{4}\right)}^{*}\right)+\frac{c-1}{2}E\left(W_{i\left(3\left(\frac{m+1}{4}\right)\right)}^{*}\right)+E\left(W_{i\left(\frac{m+1}{2}\right)}^{*}\right)+\frac{m-c-1}{2}E\left(W_{i\left(\;1:m\right)}^{*}\right)+\frac{m-c-1}{2}E\left(W_{i\left(m,m\right)}^{*}\right)+E\left(W_{m\left(1,m\right)}^{*}\right)\right)$$

where $\left(W_{i\left(\frac{m+1}{4}\right)}^{*}\right)=\mu_{i\left(\frac{m+1}{4}\right)}^{*}$ , $E\left(W_{i\left(3\left(\frac{m+1}{4}\right)\right)}^{*}\right)=\mu_{i\left(3\left(\frac{m+1}{4}\right)\right)}^{*}$, $E\left(W_{i\left(\frac{m+1}{2}\right)}^{*}\right)=\mu_{i\left(\frac{m+1}{2}\right)}^{*}E\left(W_{i\left(\;1,m\right)}^{*}\right)=\mu_{i\left(\;1,m\right)}^{*}$, $E\left(W_{i\left(m,m\right)}^{*}\right)=\mu_{i\left(m,m\right)}^{*}$ and $E\left(W_{m\left(1,m\right)}^{*}\right)={\;\mu }_{m\left(1,m\right)}^{*}$. Using symmetric condition about μ, it has ${{{\mu^{*}}_{i\left(\frac{m+1}{4}\right)}=\mu }^{*}}_{i\left(3\left(\frac{m+1}{4}\right)\right)}=\mu_{i\left(\frac{m+1}{2}\right)}^{*}=\mu_{i\left(\;1,m\right)}^{*}=\mu_{i\left(m,m\right)}^{*}={\;\mu }_{m\left(1,m\right)}^{*}=\mu^{*}$ and also $\mu^{*}=\mu$. Therefore,

$$E\left({\bar{W}}_{\left(MMQDRSS\right)}^{*}\right)=\frac{1}{mr}\;r\;\left(\frac{c-1}{2}\;\mu +\frac{c-1}{2}\mu +\mu +\frac{m-c-1}{2}\mu +\frac{m-c-1}{2}\mu +\mu \;\right)$$

$$E\left({\bar{W}}_{\left(MMQDRSS\right)}^{*}\right)=\mu$$

if m is even then the variance of ${\bar{W}}_{\left(MMQDRSS\right)}^{*}$ by using eq (3) is,

$$Var\left({\bar{W}}_{\left(MMQDRSS\right)}^{*}\right)=Var\left[\frac{1}{mr}\sum_{j=1}^{r}\left(\sum_{i=1}^{\frac{c-1}{2}}W_{i\left(\frac{m+1}{4}\right)}^{*}+\sum_{i=\frac{c-1}{2}+1}^{c}W_{i\left(3\left(\frac{m+1}{4}\right)\right)}^{*}+W_{i\left(\frac{m+1}{2}\right)}^{*}+\sum_{i=c+1}^{\frac{m-c-1}{2}}W_{i\left(\;1,m\right)}^{*}+\sum_{i=\frac{m-c-1}{2}+1}^{m-1}W_{i\left(m,m\right)}^{*}+W_{m\left(1,m\right)}^{*}\right)\right]$$

(4) $$Var\left({\bar{W}}_{\left(MMQDRSS\right)}^{*}\right)=\frac{1}{m^{2}\;r}\left(\frac{c-1}{2}\;Var\;\left(W_{i\left(\frac{m+1}{4}\right)}^{*}\right)+\frac{c-1}{2}Var\left(W_{i\left(3\left(\frac{m+1}{4}\right)\right)}^{*}\right)+Var\left(W_{i\left(\frac{m+1}{2}\right)}^{*}\right)+\frac{m-c-1}{2}Var\left(W_{i\left(\;1,m\right)}^{*}\right)+\frac{m-c-1}{2}Var\left(W_{i\left(m,m\right)}^{*}\right)+Var\left(W_{m\left(1,m\right)}^{*}\right)\right)$$

3. When both m and c are odd, m-c is even

**Example**

For *m* = 7, *c* = 3, and m-c = 4, the MMQDRSS can be selected as follows.

To select an MMQDRSS of size n=7 for r=1(m=7,c=3), identify $m^{3}=343$ (7 sets of 49 sampling units each). Consider, $Z_{i\left(j\right)k}$ become $j^{th}$ the lowest ranked unit from $i^{th}$ subsection of the set $k^{th}$, in which i,j,k=1,2,3,…,7. Order the units in each subset of the five sets based on the variable being studied.

(K) $$\begin{array}{c} \begin{array}{c} \left[\begin{array}{c} W_{11\left(1\right)}W_{12\left(1\right)}W_{13\left(1\right)3\;}W_{14\left(1\right)}W_{15\left(1\right)}W_{16\left(1\right)}W_{17\left(1\right)}\\ W_{21\left(1\right)}W_{22\left(1\right)}W_{23\left(1\right)}W_{24\left(1\right)}W_{25\left(1\right)}W_{26\left(1\right)}W_{27\left(1\right)}\\ W_{31\left(1\right)}{\;W}_{32\left(1\right)}W_{33\left(1\right)}{\;W}_{34\left(1\right)}W_{35\left(1\right)}W_{36\left(1\right)}W_{37\left(1\right)}\\ W_{41\left(1\right)}W_{42\left(1\right)}W_{43\left(1\right)}W_{44\left(1\right)}W_{45\left(1\right)\;}W_{46\left(1\right)}W_{47\left(1\right)}\\ W_{51\left(1\right)}W_{52\left(1\right)}W_{53\left(1\right)}{W\;}_{54\left(1\right)\;}W_{55\left(1\right)}W_{56\left(1\right)}W_{57\left(1\right)}\\ W_{61\left(1\right)}W_{62\left(1\right)}W_{63\left(1\right)}W_{64\left(1\right)\;}W_{65\left(1\right)}W_{66\left(1\right)}W_{67\left(1\right)}\\ W_{71\left(1\right)}W_{72\left(1\right)}W_{73\left(1\right)}W_{74\left(1\right)\;}{W\;}_{75\left(1\right)}W_{76\left(1\right)}W_{77\left(1\right)} \end{array}\right]\\ \left[\begin{array}{c} W_{11\left(2\right)}W_{12\left(2\right)}W_{13\left(2\right)}W_{14\left(2\right)}W_{15\left(2\right)}W_{16\left(2\right)}W_{17\left(2\right)}\\ W_{21\left(2\right)}W_{22\left(2\right)}W_{23\left(2\right)}W_{24\left(2\right)}W_{25\left(2\right)}W_{26\left(2\right)}W_{27\left(2\right)}\\ W_{31\left(2\right)}{\;W}_{32\left(2\right)}W_{33\left(2\right)}{\;W}_{34\left(2\right)}W_{35\left(2\right)}W_{36\left(2\right)}W_{37\left(2\right)}\\ {W_{41\left(2\right)}\;W}_{42\left(2\right)}W_{43\left(2\right)}W_{44\left(2\right)}W_{45\left(2\right)\;}W_{46\left(3\right)}W_{47\left(2\right)}\\ W_{51\left(2\right)}W_{52\left(2\right)}W_{53\left(2\right)}W_{54\left(2\right)\;}W_{55\left(2\right)}W_{56\left(2\right)}W_{57\left(2\right)}\\ W_{61\left(2\right)}W_{62\left(2\right)}W_{63\left(2\right)}W_{64\left(2\right)\;}W_{65\left(2\right)}W_{66\left(2\right)}W_{67\left(2\right)}\\ W_{71\left(2\right)}W_{72\left(2\right)}W_{73\left(2\right)}W_{74\left(2\right)}{W\;}_{75\left(2\right)}W_{76\left(2\right)}W_{77\left(2\right)} \end{array}\right]\\ \vdots \end{array}\\ \left[\begin{array}{c} W_{11\left(7\right)}W_{12\left(7\right)}W_{13\left(7\right)}W_{14\left(7\right)}W_{15\left(7\right)}W_{16\left(7\right)}W_{17\left(7\right)}\\ W_{21\left(7\right)}W_{22\left(7\right)}W_{23\left(7\right)}W_{24\left(7\right)}W_{25\left(7\right)}W_{26\left(7\right)}W_{27\left(7\right)}\\ W_{31\left(7\right)}{\;W}_{32\left(7\right)}W_{33\left(7\right)}{\;W}_{34\left(7\right)}W_{35\left(7\right)}W_{36\left(7\right)}W_{37\left(7\right)}\\ {W_{41\left(7\right)}\;W}_{42\left(7\right)}W_{43\left(7\right)}W_{44\left(7\right)}W_{45\left(7\right)}W_{46\left(7\right)}W_{47\left(7\right)}\\ W_{51\left(7\right)}W_{52\left(7\right)}W_{53\left(7\right)}W_{54\left(7\right)\;}W_{55\left(7\right)\;}W_{56\left(7\right)}W_{57\left(7\right)}\\ W_{61\left(7\right)}W_{62\left(7\right)}W_{63\left(7\right)}W_{64\left(7\right)}W_{65\left(7\right)}W_{66\left(7\right)}W_{67\left(7\right)}\\ W_{71\left(7\right)}W_{72\left(7\right)}W_{73\left(7\right)}W_{74\left(7\right)\;}{W\;}_{75\left(7\right)}W_{76\left(7\right)}W_{77\left(7\right)} \end{array}\right] \end{array}$$

Then, in each set, choose centre units in which blocks, and the units used for sampling in every set are displayed in rows from eq (K), as shown below:

$$\left[\begin{array}{c} W_{11\left(1\right)}W_{12\left(1\right)}W_{13\left(1\right)}W_{14\left(1\right)}W_{15\left(1\right)}W_{16\left(1\right)}W_{17\left(1\right)}\\ W_{21\left(2\right)}W_{22\left(2\right)}W_{23\left(2\right)}W_{24\left(2\right)}W_{25\left(2\right)}W_{26\left(2\right)}W_{27\left(2\right)}\\ W_{31\left(3\right)}W_{32\left(3\right)}W_{33\left(3\right)}W_{34\left(3\right)}W_{35\left(3\right)\;}W_{36\left(3\right)}W_{37\left(3\right)}\\ W_{41\left(4\right)}W_{42\left(4\right)}W_{43\left(4\right)}W_{44\left(4\right)}W_{45\left(4\right)}W_{46\left(4\right)}W_{47\left(4\right)}\\ W_{51\left(5\right)}W_{52\left(5\right)}W_{53\left(5\right)}W_{54\left(5\right)}W_{55\left(5\right)}W_{56\left(5\right)}W_{57\left(5\right)}\\ W_{61\left(6\right)}W_{62\left(6\right)}W_{63\left(6\right)}W_{64\left(6\right)}W_{65\left(6\right)}W_{66\left(6\right)}W_{67\left(6\right)}\\ W_{71\left(7\right)}W_{72\left(7\right)}W_{73\left(7\right)}W_{74\left(7\right)}W_{75\left(7\right)}W_{76\left(7\right)}W_{77\left(7\right)} \end{array}\right]$$

with no determining the real measurement of these sub-section units, sort a number of each subsection the preceding set once more. Sub-sequent, select the ${\left(\frac{m+1}{4}\right)}^{th}$ a ranked unit (in boxes), $W_{i\left(1:3\right)}^{*}$ to the $i^{th}$ sub-section (i=1,2,3) and select extremes unit of rank (in boxes), i.e., $W_{i\left(4:7\right)}^{*}$ to $i^{th}$ sub-section (i=4,5,6,7) actual estimation is listed below:

$$\left[\begin{array}{c} W_{1:1}^{*}\;W_{1:2}^{*}W_{1:3}^{*}W_{1:4}^{*}W_{1:5}^{*}W_{1:6}^{*}W_{1:7}^{*}\\ W_{2:1}^{*}W_{2:2}^{*}W_{2:3}^{*}W_{2:4}^{*}W_{2:5}^{*}{W_{2:6}^{*}W}_{2:7}^{*}\\ W_{3:1}^{*}W_{3:2}^{*}W_{3:3}^{*}W_{3:4}^{*}W_{3:5}^{*}W_{3:6}^{*}W_{3:7}^{*}\\ W_{4;1}^{*}W_{4:2}^{*}W_{4:3}^{*}W_{4:4}^{*}W_{4:5}^{*}W_{4:6}^{*}W_{4:7}^{*}\\ W_{5:1}^{*}W_{5:2}^{*}W_{5:3}^{*}W_{5:4}^{*}W_{5:5}^{*}W_{5:6}^{*}W_{5:7}^{*}\\ W_{6:1}^{*}W_{6:2}^{*}W_{6:3}^{*}W_{6:4}^{*}W_{6:5}^{*}W_{6:6}^{*}W_{6:7}^{*}\\ W_{7:1}^{*}W_{7:2}^{*}W_{7:3}^{*}W_{7:4}^{*}W_{7:5}^{*}W_{7:6}^{*}W_{7:7} \end{array}\right]$$

The units $\left\{W_{1\left(2\right)}^{*},W_{2\left(6\right)}^{*},W_{3\left(4\right)}^{*},W_{4\left(1\right)}^{*},W_{5\left(1\right)}^{*},W_{6\left(7\right)}^{*},W_{7\left(7\right)}^{*}\right\}$ in boxes represent MMQDRSS of size n=7. The mean of these six sampling units is defined population mean estimator as:

$${\bar{W}}_{\left(MMQDRSS\right)}^{*}=\frac{\left\{W_{1\left(2\right)}^{*}+W_{2\left(6\right)}^{*}+W_{3\left(4\right)}^{*}+W_{4\left(1\right)}^{*}+W_{5\left(1\right)}^{*}+W_{6\left(7\right)}^{*}+W_{7\left(7\right)}^{*}\right\}}{7}$$

Consider the point that, $W_{1},W_{2},\ldots ,W_{n}$ be an n random sample using a density function distribution a densityfunction $f_{W}$, functionofdistribution $F_{W}$, the mean is μ, thus, the variance is $\sigma^{2}$. The SRS indicates mean is that, ${\bar{W}}_{SRS}=\sum_{i=1}^{n}W_{i}/n$ and $E\left({\bar{W}}_{SRS}\right)=\mu$ along with $Var\left({\bar{W}}_{SRS}\right)=\sigma^{2}/n$. In this research, the cycle is repeated once, i.e., r=1. Consider $W_{i\left(\frac{m+1}{4}\right)}^{*}$ displays firstquartileunit from the $i^{th}$ sub-section $\left(i=1,2,\ldots ,\frac{c}{2}\right)$ and $W_{i\;\left(3\left(\frac{m+1}{4}\right)\right)}^{*}$ displays that, thirdquartileunit from the $i^{th}$ sub-section $\left(i=\frac{c-1}{2}+1,\ldots ,c\right)$, $Z_{i\left(\frac{m+1}{2}\right)}^{*}$ shows the median unit from the $i^{th}$ subset of $\left(i=c\right),W_{i\left(\;1:m\right)}^{*}$ and $W_{i\left(m:m\right)}^{*}$ shows the selected unites of extreme rank set sampling (even) from the $i^{th}$ subset of m−c.

The purposed estimator as:

(5) $${\bar{W}}_{\left(MMQDRSS\right)}^{*}=\frac{1}{mr}\sum_{j=1}^{r}\left(\sum_{i=1}^{\frac{c-1}{2}}W_{i\left(\frac{m+1}{4}\right)}^{*}+\sum_{i=\frac{c-1}{2}+1}^{c}W_{i\left(3\left(\frac{m+1}{4}\right)\right)}^{*}+W_{i\left(\frac{m+1}{2}\right)}^{*}+\sum_{i=c+1}^{\frac{m-c-1}{2}}W_{i\left(\;1,m\right)}^{*}+\sum_{i=\frac{m-c-1}{2}+1}^{m-1}W_{i\left(m,m\right)}^{*}\right)$$

#### 3.1. Theorem

${\bar{W}}_{\left(MMQDRSS\right)}^{*}$ is an unbiased population mean estimator.

**Proof:**

Let m is even, apply expectation on both sides of eq (5),

$$E\left({\bar{W}}_{\left(MMQDRSS\right)}^{*}\right)=E\left[\frac{1}{mr}\sum_{j=1}^{r}\left(\sum_{i=1}^{\frac{c-1}{2}}W_{i\left(\frac{m+1}{4}\right)}^{*}+\sum_{i=\frac{c-1}{2}+1}^{c}W_{i\left(3\left(\frac{m+1}{4}\right)\right)}^{*}+W_{i\left(\frac{m+1}{2}\right)}^{*}+\sum_{i=c+1}^{\frac{m-c-1}{2}}W_{i\left(\;1,m\right)}^{*}+\sum_{i=\frac{m-c-1}{2}+1}^{m-1}W_{i\left(m,m\right)}^{*}\right)\right]\text{.}$$

$$E\left({\bar{W}}_{\left(MMQDRSS\right)}^{*}\right)=\frac{1}{mr}\;r\;\left(\frac{c-1}{2}\;E\;\left(W_{i\left(\frac{m+1}{4}\right)}^{*}\right)+\frac{c-1}{2}E\left(W_{i\left(3\left(\frac{m+1}{4}\right)\right)}^{*}\right)+E\left(W_{i\left(\frac{m+1}{2}\right)}^{*}\right)+\frac{m-c}{2}E\left(W_{i\left(\;1,m\right)}^{*}\right)+\frac{m-c}{2}E\left(W_{i\left(m,m\right)}^{*}\right)\right)$$

where $\left(W_{i\left(\frac{m+1}{4}\right)}^{*}\right)=\mu_{i\left(\frac{m+1}{4}\right)}^{*}$ , $E\left(W_{i\left(3\left(\frac{m+1}{4}\right)\right)}^{*}\right)=\mu_{i\left(3\left(\frac{m+1}{4}\right)\right)}^{*}$ , $E\left(W_{i\left(\frac{m+1}{2}\right)}^{*}\right)=\mu_{i\left(\frac{m+1}{2}\right)}^{*}E\left(W_{i\left(\;1,m\right)}^{*}\right)=\mu_{i\left(\;1,m\right)}^{*}$ and $E\left(W_{i\left(m,m\right)}^{*}\right)=\mu_{i\left(m,m\right)}^{*}$ . Using symmetric condition about μ, it has ${{{\mu^{*}}_{i\left(\frac{m+1}{4}\right)}=\mu }^{*}}_{i\left(3\left(\frac{m+1}{4}\right)\right)}=\mu_{i\left(\frac{m+1}{2}\right)}^{*}=\mu_{i\left(\;1,m\right)}^{*}=\mu_{i\left(m,m\right)}^{*}=\mu^{*}$ and also $\mu^{*}=\mu$. Therefore,

$$E\left({\bar{W}}_{\left(MMQDRSS\right)}^{*}\right)=\frac{1}{mr}\;r\;\left(\frac{c-1}{2}\;\mu +\frac{c-1}{2}\mu +\mu +\frac{m-c}{2}\mu +\frac{m-c}{2}\mu \;\right)$$

$$E\left({\bar{W}}_{\left(MMQDRSS\right)}^{*}\right)=\mu$$

if m is even then the variance of ${\bar{W}}_{\left(MMQDRSS\right)}^{*}$ by using eq (5) is,

$$Var\left({\bar{W}}_{\left(MMQDRSS\right)}^{*}\right)=Var\left[\frac{1}{mr}\sum_{j=1}^{r}\left(\sum_{i=1}^{\frac{c-1}{2}}W_{i\left(\frac{m+1}{4}\right)}^{*}+\sum_{i=\frac{c-1}{2}+1}^{c}W_{i\left(3\left(\frac{m+1}{4}\right)\right)}^{*}+W_{i\left(\frac{m+1}{2}\right)}^{*}+\sum_{i=c+1}^{\frac{m-c-1}{2}}W_{i\left(\;1,m\right)}^{*}+\sum_{i=\frac{m-c-1}{2}+1}^{m-1}W_{i\left(m,m\right)}^{*}\right)\right]$$

(7) $$Var\left({\bar{W}}_{\left(MMQDRSS\right)}^{*}\right)=\frac{1}{m^{2}\;r}\left(\frac{c-1}{2}\;Var\;\left(W_{i\left(\frac{m+1}{4}\right)}^{*}\right)+\frac{c-1}{2}Var\left(W_{i\left(3\left(\frac{m+1}{4}\right)\right)}^{*}\right)+Var\left(W_{i\left(\frac{m+1}{2}\right)}^{*}\right)+\frac{m-c}{2}Var\left(W_{i\left(\;1,m\right)}^{*}\right)+\frac{m-c}{2}Var\left(W_{i\left(m,m\right)}^{*}\right)\right)$$

4. When m is an odd number, c is an even number, and m-c is an odd number

**Example:**

The MMQDRSS can be chosen as follows for m=5 and c=2.

To select an MMQDRSS of size n=5 for r=1(m=5,c=2), identify $m^{3}=125$ (5 sets of 25 sampling units each). Consider, $Z_{i\left(j\right)k}$ become $j^{th}$ the lowest ranked unit from $i^{th}$ sub-section of the set $k^{th}$, in which i,j,k=1,2,3,…,5. Order the units in each subset of the five sets based on the variable being studied.

(L) $$\begin{array}{c} \begin{array}{c} \left[\begin{array}{c} W_{11\left(1\right)}W_{12\left(1\right)}W_{13\left(1\right)}W_{14\left(1\right)}W_{15\left(1\right)}\\ W_{21\left(1\right)}W_{22\left(1\right)}W_{23\left(1\right)}W_{24\left(1\right)}W_{25\left(1\right)}\\ {\;W}_{31\left(1\right)}W_{32\left(1\right)}{\;W}_{33\left(1\right)}W_{34\left(1\right)}W_{35\left(1\right)}\\ W_{41\left(1\right)}W_{42\left(1\right)}W_{43\left(1\right)}W_{44\left(1\right)\;}W_{45\left(1\right)}\\ W_{51\left(1\right)}W_{52\left(1\right)}W_{53\left(1\right)}{W\;}_{54\left(1\right)}W_{55\left(1\right)} \end{array}\right]\\ \left[\begin{array}{c} W_{11\left(2\right)}W_{12\left(2\right)}W_{13\left(2\right)}W_{14\left(2\right)}W_{15\left(2\right)}\\ W_{21\left(2\right)}W_{22\left(2\right)}W_{23\left(2\right)}W_{24\left(2\right)}W_{25\left(2\right)}\\ {\;W}_{31\left(2\right)}W_{32\left(2\right)}{\;W}_{33\left(2\right)}W_{34\left(2\right)}W_{35\left(2\right)}\\ W_{41\left(2\right)}W_{42\left(2\right)}W_{43\left(2\right)}W_{44\left(2\right)\;}W_{45\left(2\right)}\\ W_{51\left(2\right)}W_{52\left(2\right)}W_{53\left(2\right)}W_{54\left(2\right)\;}W_{55\left(2\right)} \end{array}\right]\\ \vdots \end{array}\\ \left[\begin{array}{c} W_{11\left(5\right)}W_{12\left(5\right)}W_{13\left(5\right)}W_{14\left(5\right)}W_{15\left(5\right)}\\ W_{21\left(5\right)}W_{22\left(5\right)}W_{23\left(5\right)}W_{24\left(5\right)}W_{25\left(5\right)}\\ {\;W}_{31\left(5\right)}W_{32\left(5\right)}{\;W}_{33\left(5\right)}W_{34\left(5\right)}W_{35\left(5\right)}\\ W_{41\left(5\right)}W_{42\left(5\right)}W_{43\left(5\right)}W_{44\left(5\right)\;}W_{45\left(5\right)}\\ W_{51\left(5\right)}W_{52\left(5\right)}W_{53\left(5\right)}W_{54\left(5\right)\;}W_{55\left(5\right)} \end{array}\right] \end{array}$$

Then, from set c, select the middle units in the boxes and get average of these middle units, each set of sampling units are listed in rows from eq (L), as follows:

$$\left[\begin{array}{c} W_{11\left(1\right)}W_{21\left(1\right)}W_{13\left(1\right)}W_{14\left(1\right)}W_{15\left(1\right)}\\ W_{21\left(2\right)}W_{22\left(2\right)}W_{23\left(2\right)}W_{24\left(2\right)}W_{25\left(2\right)}\\ W_{31\left(3\right)}W_{32\left(3\right)}W_{33\left(3\right)}W_{34\left(3\right)}W_{35\left(3\right)}\\ W_{41\left(4\right)}W_{42\left(4\right)}W_{43\left(4\right)}W_{44\left(4\right)}W_{45\left(4\right)}\\ W_{51\left(5\right)}W_{52\left(5\right)}W_{53\left(5\right)}W_{54\left(5\right)}W_{55\left(5\right)} \end{array}\right]$$

with no determining the real measurement of these sub-section units, sort the elements of each subsection of the preceding once more set. Sub-sequent, select the ${\left(\frac{m+1}{4}\right)}^{th}$ a ranked unit (in boxes), $W_{i\left(1:2\right)}^{*}$ to the $i^{th}$ sub-section (i=1,2) and select extremes unit of rank (in boxes), i.e., $W_{i\left(3:5\right)}^{*}$ to $i^{th}$ sub-section (i=4,5,) actual estimation is listed below:

$$\left[\begin{array}{c} W_{1:1}^{*}\;W_{1:2}^{*}\;W_{1:3}^{*}W_{1:4}^{*}W_{1:5}^{*}\\ W_{2:1}^{*}W_{2:2}^{*}\;W_{2:3}^{*}\;W_{2:4}^{*}W_{2:5}^{*}\\ \;W_{3:1}^{*}\;W_{3:2}^{*}W_{3:3}^{*}W_{3:4}^{*}W_{3:5}^{*}\\ W_{4:1}^{*}W_{4:2}^{*}W_{4:3}^{*}W_{4:4}^{*}W_{4:5}^{*}\\ W_{5:1}^{*}W_{5:2}^{*}W_{5:3}^{*}W_{5:4}^{*}W_{5:5} \end{array}\right]$$

The units $\left\{W_{1\left(2\right)}^{*},W_{2\left(4\right)}^{*},W_{3\left(1\right)}^{*},W_{4\left(5\right)}^{*},\frac{1}{2}\left(W_{5\left(1\right)}^{*}+W_{5\left(5\right)}^{*}\right)\right\}$ in boxes represent MMQDRSS of size n=5. The mean of these six sampling units is defined population mean estimator as:

$${\bar{W}}_{\left(MMQDRSS\right)}^{*}=\frac{\left\{W_{1\left(2\right)}^{*}+W_{2\left(4\right)}^{*}+W_{3\left(1\right)}^{*}+W_{4\left(5\right)}^{*}+\frac{1}{2}\left(W_{5\left(1\right)}^{*}+W_{5\left(5\right)}^{*}\right)\right\}}{5}$$

Consider the point that, $W_{1},W_{2},\ldots ,W_{n}$ be an n random sample using a density function distribution with a densityfunction $f_{W}$, functionofdistribution $F_{W}$, the mean is μ and the variance is $\sigma^{2}$. The SRS indicates mean is that, ${\bar{W}}_{SRS}=\sum_{i=1}^{n}W_{i}/n$ and $E\left({\bar{W}}_{SRS}\right)=\mu$ along with $Var\left({\bar{W}}_{SRS}\right)=\sigma^{2}/n$. In this research, the cycle is repeated once, i.e., r=1. Consider $W_{i\left(\frac{m+1}{4}\right)}^{*}$ displays the firstquartileunit the $i^{th}$ sub-section $\left(i=1,2,\ldots ,\frac{c}{2}\right)$ and $W_{i\;\left(3\left(\frac{m+1}{4}\right)\right)}^{*}$ displays the third quartile unit from the $i^{th}$ sub-section $\left(i=\frac{c}{2}+1,\ldots ,c\right)$, $W_{i\left(\;1:m\right)}^{*}$, $W_{i\left(m:m\right)}^{*}$ and $W_{m\left(1:m\right)}^{*}$ shows the selected unites of extreme rank set sampling (odd) from the $i^{th}$ subset of m−c.

The purposed estimator as:

(8) $${\bar{W}}_{\left(MMQDRSS\right)}^{*}=\frac{1}{mr}\sum_{j=1}^{r}\left(\sum_{i=1}^{\frac{c}{2}}W_{i\left(\frac{m+1}{4}\right)}^{*}+\sum_{i=\frac{c}{2}+1}^{c}W_{i\left(3\left(\frac{m+1}{4}\right)\right)}^{*}+\sum_{i=c+1}^{\frac{m-c-1}{2}}W_{i\left(\;1,m\right)}^{*}+\sum_{i=\frac{m-c-1}{2}+1}^{m-1}W_{i\left(m,m\right)}^{*}+W_{m\left(1,m\right)}^{*}\right)$$

#### 1.4. Theorem

${\bar{Z}}_{\left(MMQDRSS\right)}^{*}$ is an unbiased population mean estimator.

**Proof:**

Let m is even, apply expectation on both sides of eq (8),

$$E\left({\bar{W}}_{\left(MMQDRSS\right)}^{*}\right)=E\left[\frac{1}{mr}\sum_{j=1}^{r}\left(\sum_{i=1}^{\frac{c}{2}}W_{i\left(\frac{m+1}{4}\right)}^{*}+\sum_{i=\frac{c}{2}+1}^{c}W_{i\left(3\left(\frac{m+1}{4}\right)\right)}^{*}+\sum_{i=c+1}^{\frac{m-c-1}{2}}W_{i\left(\;1:m\right)}^{*}+\sum_{i=\frac{m-c-1}{2}+1}^{m-1}W_{i\left(m:m\right)}^{*}+W_{m\left(1:m\right)}^{*}\right)\right]\text{.}$$

$$E\left({\bar{W}}_{\left(MMQDRSS\right)}^{*}\right)=\frac{1}{mr}\;r\;\left(\frac{c}{2}\;E\;\left(W_{i\left(\frac{m+1}{4}\right)}^{*}\right)+\frac{c}{2}E\left(W_{i\left(3\left(\frac{m+1}{4}\right)\right)}^{*}\right)+\frac{m-c-1}{2}E\left(W_{i\left(1,m\right)}^{*}\right)+\frac{m-c-1}{2}E\left(W_{i\left(m,m\right)}^{*}\right)+E\left(W_{m\left(1,m\right)}^{*}\right)\right)$$

where $\left(W_{i\left(\frac{m+1}{4}\right)}^{*}\right)=\mu_{i\left(\frac{m+1}{4}\right)}^{*}$ , $E\left(W_{i\left(3\left(\frac{m+1}{4}\right)\right)}^{*}\right)=\mu_{i\left(3\left(\frac{m+1}{4}\right)\right)}^{*}$ , $E\left(W_{i\left(\;1,m\right)}^{*}\right)=\mu_{i\left(\;1,m\right)}^{*}$, $E\left(W_{i\left(m,m\right)}^{*}\right)=\mu_{i\left(m,m\right)}^{*}$ and $E\left(W_{m\left(1,m\right)}^{*}\right)={\;\mu }_{m\left(1,m\right)}^{*}$. Using symmetric condition about μ, it has ${{{\mu^{*}}_{i\left(\frac{m+1}{4}\right)}=\mu }^{*}}_{i\left(3\left(\frac{m+1}{4}\right)\right)}=\mu_{i\left(\;1,m\right)}^{*}=\mu_{i\left(m,m\right)}^{*}={\;\mu }_{m\left(1,m\right)}^{*}=\mu^{*}$ and also $\mu^{*}=\mu$. Therefore,

$$E\left({\bar{W}}_{\left(MMQDRSS\right)}^{*}\right)=\frac{1}{mr}\;r\;\left(\frac{c}{2}\;\mu +\frac{c}{2}\mu +\frac{m-c-1}{2}\mu +\frac{m-c-1}{2}\mu +\mu \;\right)$$

$$E\left({\bar{W}}_{\left(MMQDRSS\right)}^{*}\right)=\mu$$

if m is even then the variance of ${\bar{W}}_{\left(MMQDRSS\right)}^{*}$ by using eq (8) is,

$$Var\left({\bar{W}}_{\left(MMQDRSS\right)}^{*}\right)=Var\left[\frac{1}{mr}\sum_{j=1}^{r}\left(\sum_{i=1}^{\frac{c}{2}}W_{i\left(\frac{m+1}{4}\right)}^{*}+\sum_{i=\frac{c}{2}+1}^{c}W_{i\left(3\left(\frac{m+1}{4}\right)\right)}^{*}+\sum_{i=c+1}^{\frac{m-c-1}{2}}W_{i\left(\;1,m\right)}^{*}+\sum_{i=\frac{m-c-1}{2}+1}^{m-1}W_{i\left(m,m\right)}^{*}+W_{m\left(1,m\right)}^{*}\right)\right]$$

(9) $$Var\left({\bar{W}}_{\left(MMQDRSS\right)}^{*}\right)=\frac{1}{m^{2}\;r}\left(\frac{c}{2}\;Var\;\left(W_{i\left(\frac{m+1}{4}\right)}^{*}\right)+\frac{c}{2}Var\left(W_{i\left(3\left(\frac{m+1}{4}\right)\right)}^{*}\right)+\frac{m-c-1}{2}Var\left(W_{i\left(\;1,m\right)}^{*}\right)+\frac{m-c-1}{2}Var\left(W_{i\left(m,m\right)}^{*}\right)+Var\left(W_{m\left(1,m\right)}^{*}\right)\right)$$
